# Neurophysiological Testing is Useful for the Diagnosis and Treatment of Neuropsychiatric Systemic Lupus Erythematosus

**DOI:** 10.1155/crpe/8637806

**Published:** 2026-06-29

**Authors:** Erika Hidawa, Moe Yoshimura, Takuya Endo, Hideo Yamanouchi, Yuko Akioka

**Affiliations:** ^1^ Department of Pediatrics, Saitama Medical University, 38 Morohongo Moroyama Iruma, Saitama, 350-0495, Japan, saitama-med.ac.jp

## Abstract

Systemic lupus erythematosus (SLE) is a chronic autoimmune disease characterized by multiorgan damage mediated by multiple autoantibodies. The nervous system is one of the target organs of SLE, presenting as neuropsychiatric SLE (NPSLE), which includes central nervous system (CNS) involvement, peripheral neuropathy, and psychiatric symptoms. These symptoms are not necessarily associated with systemic disease activity and are often challenging to diagnose. We report an 11‐year‐old girl presenting with persistent fever and mild headache. Based on clinical and laboratory findings, she was diagnosed with SLE complicated by antiphospholipid antibody syndrome. Brain magnetic resonance imaging revealed multiple small lesions in the bilateral deep white matter of the frontal lobes, with high signal intensity on diffusion‐weighted imaging and low signal intensity on apparent diffusion coefficient maps, consistent with multiple cerebral infarcts. Although multiple cerebral infarcts were present in this case, no localized clinical symptoms corresponding to the infarct locations were observed, and the case did not meet the definition of NPSLE. Conversely, electroencephalography (EEG) revealed diffuse high‐amplitude slow waves in the occipital region, and short‐latency somatosensory evoked potentials (SSEPs) showed absence of the N20 component at C3’ following right median nerve stimulation. These neurophysiological findings supported a comprehensive diagnosis of CNS NPSLE. These abnormalities gradually improved during immunosuppressive treatment. NPSLE represents one of the most challenging organ‐specific manifestations to diagnose in pediatric SLE due to its complex and heterogeneous clinical presentation and the absence of standardized diagnostic screening methods. Unlike lupus nephritis, complement consumption is not necessarily involved in the pathogenesis of NPSLE. Therefore, NPSLE may remain undiagnosed unless clinically suspected. In this case, abnormal EEG findings and absence of the N20 component on SSEP suggested ischemic changes due to cerebrovascular disease, enabling a comprehensive diagnosis of CNS NPSLE. Neurophysiological testing proved useful for early diagnosis of NPSLE and evaluation of treatment efficacy.

## 1. Introduction

Systemic lupus erythematosus (SLE) is a systemic autoimmune disease characterized by the production of multiple autoantibodies, leading to immune complex formation and subsequent deposition in various organs, resulting in multiorgan damage. The nervous system is one of the target organs in SLE, and manifestations encompass central and peripheral neurologic syndromes, as well as psychiatric syndromes. This condition is known as neuropsychiatric SLE (NPSLE) [1]. The American College of Rheumatology (ACR) developed case definitions for 19 neuropsychiatric syndromes in SLE [1]. These definitions are also applicable to juvenile‐onset SLE. NPSLE is a severe form of the disease that affects approximately 25% of pediatric SLE cases [2, 3]. However, because symptoms are diverse and multiple disease types may overlap, diagnosis can be difficult in some cases [4]. We report a case of pediatric SLE in which the patient presented with mild headaches and was found to have multiple cerebral infarcts with positive antiphospholipid antibodies. Despite the absence of focal neurological symptoms corresponding to the infarct locations and not meeting the diagnostic criteria for NPSLE, the diagnosis was established based on findings from neurophysiological testing.

## 2. Case Presentation

**FIGURE 1:**
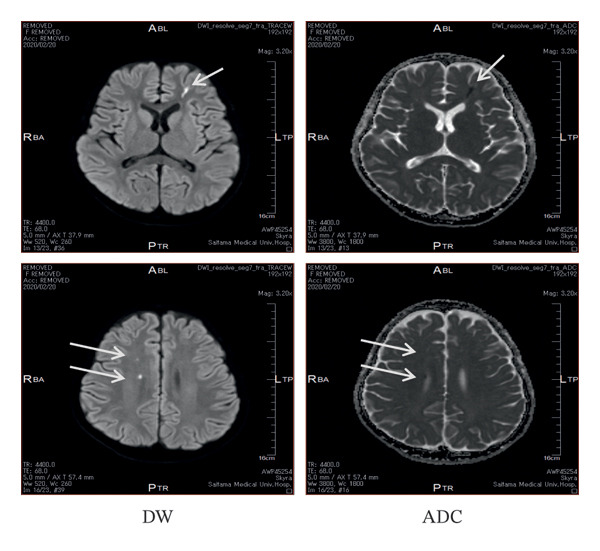
Pretreatment brain magnetic resonance imaging examination revealed high signal intensity areas in the bilateral white matter of the frontal lobe on diffusion‐weighted imaging and low signal intensity areas in the same region on the apparent diffusion coefficient map.

An 11‐year‐old girl was admitted with persistent fever for > 19 days and mild headache for 10 days. Her medical and family histories were unremarkable. On admission, she was febrile (40.0°C) and tachycardic (132/min). Her blood pressure was 95/50 mmHg. Physical examination revealed a butterfly rash on her face and arthralgia affecting both shoulders, hands, and knees. Her behavior was cooperative, and her mental status was stable. Neurological examination findings were normal, except for a persistent nonlocalized headache. Blood tests revealed lymphopenia (white blood cell count = 719/μL), thrombocytopenia (platelet count = 71,000/μL), an elevated C‐reactive protein (CRP) level (0.80 mg/L), and an elevated erythrocyte sedimentation rate (81 mm/h). Urinalysis results were normal. Immunological examination revealed elevated Immunoglobulin G (2016 mg/dL); low C3 and C4 levels; and increased antinuclear antibodies (at a titer of 1:320), anti‐dsDNA antibodies, and anti‐Sm antibodies. As these findings satisfied the SLE diagnostic criteria of the European League Against Rheumatism (EULAR)/ACR, the patient was diagnosed with SLE [5]. Lupus anticoagulant (dilute Russell’s viper venom time) and anticardiolipin antibodies IgG (enzyme‐linked immunosorbent assay) were positive, and partial thromboplastin time was prolonged. The patient was suspected of having antiphospholipid antibody syndrome (APS) superimposed on SLE. Brain magnetic resonance imaging (MRI) revealed multiple small lesions with high signal intensity on diffusion‐weighted imaging and low signal intensity on apparent diffusion coefficient maps of the deep white matter of both frontal lobes (Figure 1).

**FIGURE 2:**
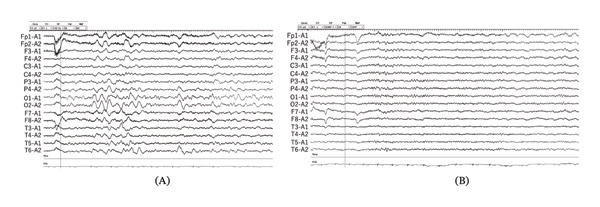
(A) Pretreatment electroencephalography (EEG) showed predominantly slow basic activity with 5–6 Hz theta waves mixed with 2.5 Hz high‐voltage delta waves in the occipital area. (B) Posttreatment EEG showed normalized basic activity with 9–10 Hz alpha waves.

**FIGURE 3:**
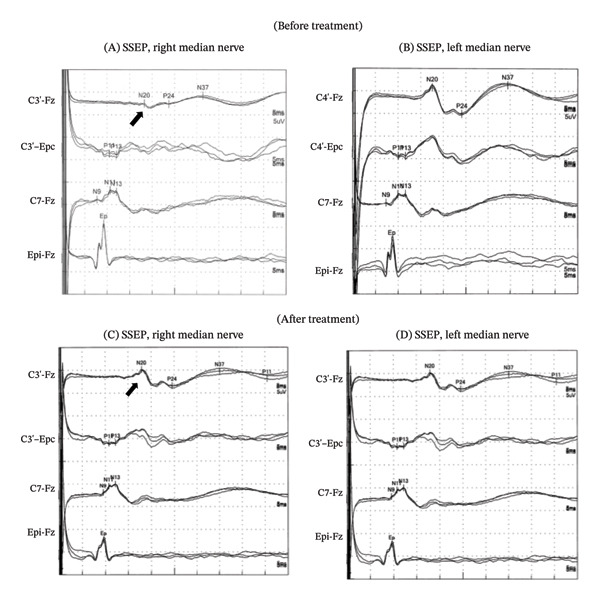
(A) In short‐latency somatosensory evoked potentials (SSEP), the N20 component was absent at C3′–Fz following right median nerve stimulation (arrow). (B) In SSEP, the N20 component was present at C3′‐Fz following left median nerve stimulation. (C) Posttreatment, in SSEP, reappearance of the N20 component with an amplitude of 1.73 μV at C3′‐Fz from baseline on the right side (arrow). (D) Posttreatment, in SSEP, the N20 component was present at C3′‐Fz following left median nerve stimulation. Note: electrode locations: Fz: the midforehead electrode international 10–20 system; C3′ and C4′: 2 cm posterior to C3 and C4; Epc: erb’s point contralateral to the stimulated limb; C7: the seventh cervical vertebra; Epi: erb’s point ipsilateral to the stimulated limb.

Based on abnormal blood test findings and multiple cerebral infarcts on brain MRI, she was diagnosed with APS. The headache, which is a symptom of NPSLE, was milder than in typical cases, and no neurological findings corresponding to the infarct locations were observed. Therefore, neurophysiological testing was performed. Electroencephalogram (EEG) showed slow basic activity with predominantly 5–6 Hz theta waves mixed with 2.5 Hz high‐voltage delta waves in the occipital area (Figure 2A). Short‐latency somatosensory evoked potentials (SSEPs) showed absence of the N20 component at C3′ following right median nerve stimulation (Figures 3A and B). The patient was diagnosed with central nervous system (CNS)–type NPSLE. Regarding cerebrospinal fluid testing, the patient did not consent.

Three courses of intravenous methylprednisolone pulse therapy (1 g/day for 3 days) were administered. On Day 26 of hospitalization, a kidney biopsy was performed in accordance with Japanese clinical practice guidance for childhood‐onset SLE [6], which suggested the possibility of asymptomatic lupus nephritis, and the patient was diagnosed with lupus nephritis (Class III). The SLE Disease Activity Index 2000 score was 19 (visual disturbance, arthritis, low complement, increased DNA binding, fever, thrombocytopenia, and leukopenia), indicating high disease activity [7]. Therefore, cyclophosphamide pulse therapy was administered four times at 4‐week intervals after methylprednisolone pulse therapy. Her symptoms improved rapidly, and her serology, including hypocomplementemia and cytopenia, gradually improved. After the three courses of intravenous methylprednisolone pulse therapy, the EEG exhibited basic activity with 8.5 Hz alpha waves. After the first course of cyclophosphamide pulse therapy, the EEG showed basic activity with 9.5 Hz alpha waves. After the second course of cyclophosphamide pulse therapy, the EEG showed normalized basic activity with 10.5 Hz alpha waves (Figure 2B). SSEP showed reappearance of the N20 component with an amplitude of 1.73 μV at C3′–Fz, and no difference in amplitude was observed between the left and right sides after the first course of cyclophosphamide pulse therapy (Figures 3C and D).

## 3. Discussion

NPSLE is one of the most challenging organ‐specific manifestations to diagnose in pediatric SLE patients. This difficulty arises from the complex and heterogeneous clinical presentations of SLE and the fact that NPSLE is often a diagnosis of exclusion because of the absence of standardized diagnostic screening methods [8]. The manifestations of NPSLE are characterized by marked variability and unpredictability. Unlike lupus nephritis, complement consumption is not necessarily involved in the pathogenesis of NPSLE [9]. Therefore, NPSLE is diagnosed only after recognizing its clinical variability and suspecting the patient’s symptoms. NPSLE manifestations are classified primarily into CNS and peripheral nervous system symptoms. CNS symptoms are further classified into focal and diffuse types [1]. Focal CNS disease corresponds to neurological symptoms, while diffuse CNS disease corresponds to psychiatric symptoms. Neurological symptoms are classified into aseptic meningitis, cerebrovascular disorders, demyelinating diseases, headache, movement disorders (chorea), myelopathy, and seizures, each with its own definition. Benseler SM et al. reported that the incidence of NPSLE in pediatric SLE patients is approximately 25%. Among pediatric SLE manifestations, headaches occur in 66%, psychiatric symptoms in 36%, cognitive impairment in 25%, and cerebrovascular disorders in 24%. Headaches, in particular, are frequently observed as an early symptom of NPSLE and are often associated with other CNS disorders, especially cerebrovascular disease diagnosed by MRI [2].

In the present case, the patient presented with multiple cerebral infarcts, positive antiphospholipid antibodies, and mild headache but lacked focal neurological symptoms, thus not meeting the diagnostic criteria. However, diffuse high‐amplitude slow waves on EEG, and the absence of the N20 component supported a comprehensive diagnosis of CNS NPSLE. MRI remains the standard tool for detecting brain and spinal cord lesions in NPSLE. However, few studies have reported the diagnostic value of neurophysiological testing in NPSLE.

EEG serves as a supplementaly diagnostic tool in NPSLE, though its clinical utility remains unclear. EEG abnormalities have been observed in 45%–71% of pediatric NPSLE cases. Although EEG is useful for evaluating patients with seizures, it demonstrates limited sensitivity for diagnosing NPSLE. The most common findings in EEG include diffuse background slowing and epileptiform discharges with either focal or generalized localization [4]. Olfat et al., in an observational study of 20 pediatric NPSLE patients, reported a high rate of background slowing on EEG in patients with cerebrovascular disorders [10], which is consistent with the EEG findings in this case. Mostafa et al. also reported EEG abnormalities in 15 of 22 patients (68.2%) with pediatric NPSLE, whereas EEG findings were normal in 8 patients without NPSLE [11]. Although diagnostic sensitivity is low, because no abnormalities are found in patients without NPSLE, EEG testing may be meaningful in pediatric SLE patients suspected of having NPSLE. EEG may serve as a useful parameter if performed during the initial evaluation, before overt NPSLE manifestations.

Furthermore, reports on evoked potentials in NPSLE are rare. SSEPs measure small electrical potentials elicited on the scalp or other areas in response to sensory stimulation of peripheral nerves and are used to estimate dysfunction or lesion localization in the medial lemniscus pathway. Saad Allah et al. found that 12 of 18 patients with NPSLE exhibited prolonged latencies in SSEP, indicating that neurophysiological testing, including SSEP, is useful for detecting minor CNS abnormalities in neurologically asymptomatic patients [12]. In the present case, the absence of the N20 wave, which originates from the left cortical sensory area, in the right median nerve stimulation SSEP findings suggested a lesion in the left thalamus and internal capsule region of the left cerebral hemisphere, consistent with left cerebral infarction. Posttreatment, these findings improved, and the asymmetry disappeared, suggesting that SSEP, along with EEG, is a valuable noninvasive test for assessing treatment efficacy in pediatric NPSLE. However, the presence of conduction abnormalities exclusively in the left hemisphere, despite multiple bilateral microinfarcts, remains unexplained.

Although a cerebrospinal fluid (CSF) examination can be helpful in the diagnosis of NPSLE [1, 5], we did not perform it due to the inability to secure the patient’s consent. This was considered a limitation that may have made the diagnosis of NPSLE more difficult.

## 4. Conclusion

In this case, multiple cerebral infarcts and positive antiphospholipid antibodies were present; however, no focal clinical symptoms corresponding to the infarct locations were observed, complicating the diagnosis. The presence of abnormal EEG findings, along with diminished SSEP amplitudes, indicated ischemic changes. Neurophysiological testing provided valuable functional evidence supporting clinical attribution and may serve as a useful adjunctive tool in the diagnosis and monitoring of pediatric NPSLE.

## Author Contributions

Erika Hidawa, Moe Yoshimura, Takuya Endo, Hideo Yamanouchi, and Yuko Akioka diagnosed and managed the patient. Erika Hidawa wrote the first draft of the manuscript. Moe Yoshimura and Takuya Endo contributed to the conception and critical revision of the manuscript. Hideo Yamanouchi and Yuko Akioka supervised the conception, drafted the manuscript, and critically revised the manuscript.

## Funding

No funding was received to support this study.

## Consent

Signed informed consent was obtained directly from the patient.

## Conflicts of Interest

The authors declare no conflicts of interest.

## Data Availability

The data that support the findings of this study are available on request from the corresponding author. The data are not publicly available due to privacy or ethical restrictions.
